# Circadian Changes in the Sputum of Asthmatic Subjects and Healthy Controls

**DOI:** 10.1097/WOX.0b013e3181752d02

**Published:** 2008-05-15

**Authors:** Todor A Popov, Mohamed S Shenkada, Anna V Tzoncheva, Maria P Pravtchanska, Tihomir B Mustakov, Vasil D Dimitrov

**Affiliations:** 1 Clinical Centre of Allergology, Medical University, Sofia, Bulgaria; 2 Clinical Laboratory, Medical University, Sofia, Bulgaria

**Keywords:** asthma, sputum, eosinophils, ECP, neutrophils, macrophages, circadian changes, cortisol

## Abstract

**Background:**

Asthma is exhibiting classical circadian fluctuations of clinical symptoms and airflow measurements, presumably influenced by the underlying airway inflammation and the endogenous cortisol secretion. The aim of our study was to examine the cellular and eosinophilic cationic protein (ECP) contents of sputum, collected and frozen at 2 opposite time points of the day-and-night cycle, and to correlate them to blood cortisol levels.

**Methods:**

Thirteen subjects with uncontrolled asthma (8 men, aged 25-54 years) and 10 healthy subjects (6 men, aged 25-50 years) volunteered for the study. They were induced with hypertonic saline to produce sputum between 8:00 AM and 9:00 AM and between 8:00 PM and 9:00 PM, when blood was also withdrawn to measure cortisol levels.

**Results:**

Asthmatic subjects did not display significant morning/evening differences in their sputum total cell counts ([TCCs] median, 2.06 × 10^3 ^cells/mL; range 0.50-5.66 cells/mL vs median, 1.29 cells/mL; range, 0.24-9.26 cells/mL, *P *> 0.1), whereas controls had a well-defined morning peak (median, 0.75 cells/mL; range, 0.31-2.25 cells/mL vs median, 0.33 cells/mL; range, 0.1-0.97 cells/mL, *P *> 0.001). Asthmatic subjects had significantly higher sputum TCC than controls in the evening (*P *< 0.001), but their morning TCC did not significantly exceed those of the healthy subjects. Asthmatic subjects had significantly more sputum eosinophils and higher ECP levels than controls but failed to demonstrate significant morning/evening differences in contrast to the controls who had higher morning eosinophils and ECP. Macrophages were relatively increased in the evening samples of both asthmatic subjects and controls. No significant correlations between the circadian cortisol shift and any of the sputum indices were found.

**Conclusions:**

Sputum undergoes circadian changes, which are different in health and in asthma and do not correlate with endogenous cortisol levels.

## 

Among the different noninvasive methods to assess airway inflammation, sputum examination has established itself as a relatively simple and less expensive approach, affordable to wider numbers of researchers and clinicians. Among its advantages is the possibility to make serial measurements to flag the time points of naturally occurring or provoked changes. However, sputum measurements made at different hours of the day and night may be influenced by circadian alterations in health or disease. This may be all the more important for bronchial asthma because its clinical course usually demonstrates pronounced circadian variations [1]. Although morning day-to-day repeatability of sputum examination in subjects with stable asthma or healthy controls has been well documented, [2-4] circadian changes of sputum indices have been the subject of only 1 original article [5]. A possible explanation of this deficit of studies are the technical difficulties related to the need to process the collected sputum within 2 hours with the standard protocols and the ensuing organizational problems when designing such round-the-clock studies.

Freezing of sputum immediately after collection for subsequent processing at the convenience of the laboratory technicians is an elegant way to circumvent the time restrictions posed by the rich enzymatic content of this body fluid, which would digest its cellular and fluid phase components if the sample is left unattended. This technology has been worked out by different research teams since the turn of the millennium [6-9]. It is logical to apply it for the assessment of sputum at time points inconvenient for direct handling. In this study, we used it to assess eventual differences in the sputum of asthmatic subjects and healthy controls at 2 extremes of the day-and-night cycle: between 8:00 AM and 9:00 AM and between 8:00 PM and 9:00 PM.

## Methods

### Subjects

Thirteen subjects (8 men, age range between 25 and 54 years) with uncontrolled asthma [10] admitted to the Allergology Clinic of Alexander's University Hospital in Sofia because of inadequate control of their disease and 10 volunteers (6 men, aged 25-50 years) from among its personnel volunteered in this cross-sectional study. The asthmatic subjects had baseline treatment of inhaled beclomethasone dipropionate at doses ranging between 500 and 1000 μg/d and inhaled salbutamol as needed. None had received oral or systemic corticosteroids within the preceding week. They all occasionally had nighttime symptoms but did not display classical features of nocturnal asthma, defined as greater than 20% fall in peak expiratory flow rate from bedtime to morning wakening [11]. None of the subjects had had signs of viral infection within the month before the measurements. The study was approved by the ethics committee of Alexander's University Hospital in Sofia.

### Laboratory Measurements

#### Blood Samples

Blood was withdrawn between 8:00 AM and 9:00 AM and between 8:00 PM and 9:00 PM, and the serum was frozen at -70°C for cortisol assessment by Chiron Diagnostics ACS:180 automated system. This is a competitive immunoassay on a solid phase using direct chemiluminescence technology. The results are expressed in nanograms per milliliter.

#### Sputum Induction

Sputum was induced between 8:00 AM and 9:00 AM and between 8:00 PM and 9:00 PM of 1 day as described before [12]. Briefly, after baseline spirometry and pretreatment with salbutamol, subjects inhaled 3, 4 and 5% hypertonic saline for 7 minutes periods. After each period they tried to cough out sputum into plastic containers.

#### Sputum Freezing and Storage

Sputum specimens were poured into Petri dishes right after induction, the portions originating from the lower airways were selected and put into Eppendorf tubes, and 100 μL of dimethyl sulfoxide (Merck, Germany) added. The tubes were then sealed, vortexed for 30 seconds, coded, and stored in a freezer at -70°C.

#### Sputum Processing

The frozen sputum samples were processed by a regular-shift laboratory assistant in the morning hours after a previously described protocol, [2] with slight modifications. The Eppendorf tubes were left at room temperature to thaw, and the weight of their content was measured. Dithiothreitol (Sputalysin, Calbiochem Corp, La Jolla, Calif) 1 to 10 diluted solution was added in a volume (in microliters) 4 times bigger than the sputum weight (in milligrams). The mixture was well homogenized by vortexing and rocking, Dulbecco phosphate-buffered saline equal in volume to the dithiothreitol was added, and the suspension was filtered. Cell viability was assessed by trypan blue exclusion, and the total cell count (TCC) per milligram of sputum was calculated on the basis of a hemocytometer count, taking into consideration the overall dilution, and the 100-μL dimethyl sulfoxide used for cryopreservation was included. The cell suspension was centrifuged, and the supernatant was stored at -70°C for subsequent determination of eosinophil cationic protein (ECP). The cell pellet was resuspended in Dulbecco phosphate-buffered saline, and cytospins were made using a Hettich Universal cytocentrifuge (Tuttlingen, Germany). They were stained with Giemsa for a differential count on 400 non-squamous cells.

The ECP in the sputum supernatants was measured by UniCAP 100 (Phadia, Uppsala, Sweden). The results are expressed in micrograms per liter.

### Data Analysis

Descriptive statistics were used to characterize the subjects in the study. Data with suspected nonnormal distribution (sputum eosinophils, neutrophils and macrophages, sputum ECP) were presented with their median and extreme values and were log-transformed before the statistical analysis. Comparisons between outcomes in asthmatic subjects and controls were made using independent sample *t *test, whereas a paired *t *test was used to evaluate morning-evening differences. The correlation between variables was examined using Spearman rank correlation coefficients. A 2-tailed probability, *P *< 0.05, was considered statistically significant. The SPSS for Windows software was used.

## Results

Asthmatic subjects and controls did not differ in age. The asthmatic subjects had significantly lower forced expiratory volume in 1 second (76.5% of predicted; range, 58%-92% vs 97.5% of predicted; range, 83%-111%) and higher blood eosinophils (mean, 558.4 cells/mL ± 94.9 cells/mL [SEM] vs mean, 146.3 cells/mL ± 92.8 cells/mL [SEM]).

All 23 subjects were able to produce adequate sputum samples upon induction.

**Figure 1:**
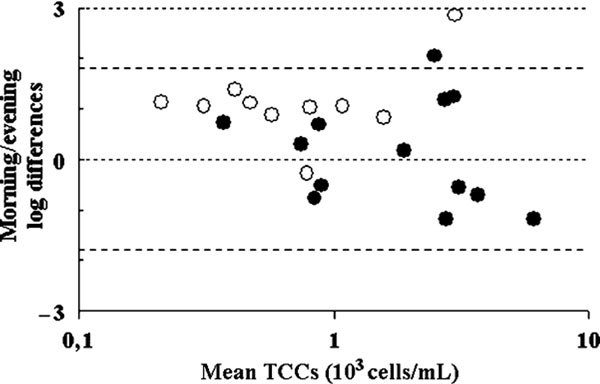
**Difference in logs between the morning and evening sputum TCCs plotted against the mean morning evening values (Bland and Altman plot)**. Open symbols represent controls, and the solid ones, asthmatic subject samples. The dashed lines are delineating an area of 2 SDs above and below the ''no-difference zero line'' of the morning-evening differences.

The morning sputum samples of the controls yielded higher numbers of cells than those in the evening: the morning median TCC was 0.75 × 10^3 ^cells/mL (range, 0.31-2.25 × 10^3 ^cells/mL) versus 0.33 × 10^3 ^cells/mL (range, 0.1-0.97 × 10^3 ^cells/mL) in the evening (*P *= 0.001). The asthmatic subjects did not display significant circadian differences in their sputum TCC, 2.06 × 10^3 ^cells/mL (range, 0.5-5.66 × 10^3 ^cells/mL) in the morning versus 1.29 × 10^3 ^cells/mL (range, 0.24-9.26 × 10^3 ^cells/mL) in the evening (*P *> 0.05). The independent sample *t *test comparing asthmatic subjects and controls revealed no difference between the log-transformed TCC of the morning sputum samples and significantly higher cell numbers in the evening samples of asthmatic subjects (*P *= 0.001). These relationships are presented graphically in Figure 1.

Cell viability was consistently higher in the morning hours for all study subjects: 73.4% ± 3.5% (mean ± SEM) viability in the morning versus 56.2% ± 2.8% viability in the evening. The morning and evening values and the morning/evening differences were similar for asthmatic subjects and healthy controls.

**Figure 2:**
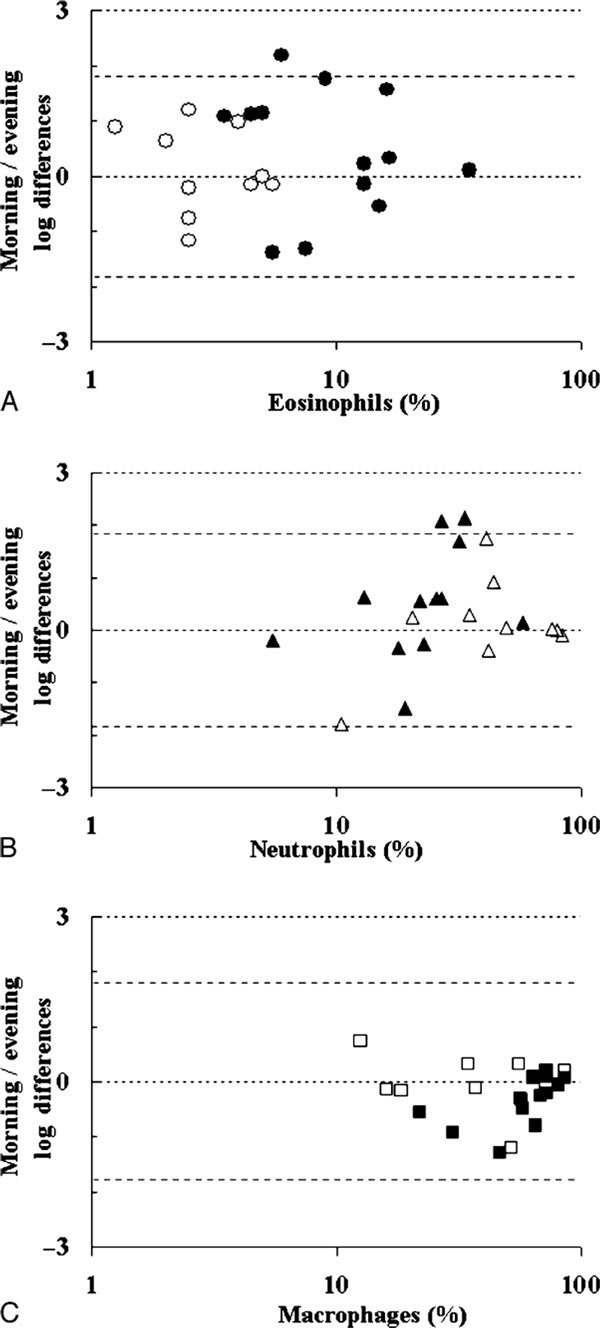
**Bland and Altman plots of the differences in logs between morning and evening sputum eosinophils (A), neutrophils (B), and macrophages (C)**. Open symbols represent controls, solid symbols, asthmatic subjects.

The sputum samples of asthmatic subjects contained significantly more eosinophils as compared with the normal controls. The healthy subjects had a significant drop in the relative and absolute eosinophil numbers in their evening samples, whereas no such difference was noted in the asthmatic sputa (Figure 2A). The morning samples of asthmatic subjects tended to have more neutrophils as compared with their evening specimens, but this trend did not reach statistical significance (*P *= 0.071; Figure 2B). Macrophages were consistently increased in the evening samples of both asthmatic subjects and controls (*P *= 0.048; Figure 2C). Lymphocytes did not show any specific circadian pattern.

**Figure 3:**
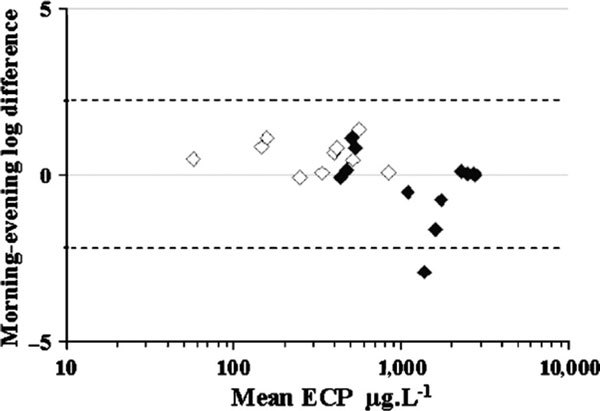
**Bland and Altman plot of the differences between morning and evening sputum ECP values**. Open symbols represent controls, solid symbols, asthmatic subjects.

Asthmatic subjects had significantly higher ECP morning and evening values than the healthy subjects (Figure 3). The morning values of the controls were significantly higher than their evening ones (*P *= 0.004), whereas the asthmatic subjects had somewhat higher values in the evening, but the level of significance was not reached.

The healthy controls had higher morning levels of serum cortisol: 396.9 nmol/L (mean) ± 61.7 nmol/L (SEM) versus 208.6 nmol/L ± 39.8 nmol/L (*P *= 0.03), whereas there were no differences between the morning and evening cortisol levels of the asthmatic subjects: 387.8 nmol/L ± 107.5 nmol/L versus 405.7 nmol/L ± 112.5 nmol/L (*P *> 0.05). The analysis did not disclose significant correlations between the morning-evening shift in the serum cortisol levels and a corresponding shift in any of the sputum indices measured.

Multiple regression analysis could not identify any associations between the clinical features of the asthmatic subjects and the circadian values of their blood cortisol and sputum indices.

## Discussion

Circadian rhythms can influence the response of patients to diagnostic tests and therapeutic interventions according to their timing with reference to body rhythms [13]. Asthma is a classical model for a circadian-modulated disease, with the term *nocturnal asthma *coined for the clinical form with exclusive night manifestations. The number of studies, relying on outcomes in sputum to assess the state of the airways in asthmatic subjects, has been increasing steadily over the past decade. Unlike bronchoalveolar lavage and bronchial biopsies, which take a "snapshot" of the cellular- and fluid-phase components of a limited segment of the airways, sputum is a product of naturally occurring events over time, bringing to the outlet (mouth) an integral mix of elements from all over the bronchial tree. Thus, sputum examination is representing a compartment different from the one assessed by biopsies and bronchoalveolar lavage [14].

During nighttime, including sleep and/or quiet wakefulness, changes in the ventilatory patterns and airway responsiveness take place [11]. These have been associated also with variations in the lung tissue inflammation as assessed by invasive bronchoscopic methods [15,16]. Panzer et al [5] have attempted to establish correlations between ventilation outcomes and inflammation of the airways on the basis of sputum indices. They have come up with conclusions about a morning peak pattern of changes in TCC, eosinophils, and eosinophil-related mediators in their set of asthmatic subjects. Their results differ from ours, so it is worthwhile to consider possible explanations for these seeming discrepancies. Whereas their study has only 1 arm of mild asthmatic subjects, we have made our sputum assessment in uncontrolled asthmatic subjects and healthy controls, and we notice distinct patterns in these 2 groups. It turns out that morning-to-evening changes in sputum are characteristic of our controls, and are rather blurred in our asthmatic population. Interestingly, this is paralleled by changes in blood cortisol levels, which are with a morning peak in the controls, showing no differences in the asthmatic subjects. It could be speculated that flourishing airway inflammation may not be subject to physiological circadian influences, which themselves may be compromised in our patients. All our asthmatic subjects were on maintenance treatment with mild to moderate doses of inhaled corticosteroids as specified in the Global Initiative for Asthma 2006 edition [10]. It could be speculated that either the inhaled corticosteroids could have some systemic effect obliterating the normal circadian functioning of the hypothalamopituitary adrenal axis, or the activity of the disease itself is interfering with it. A study comparing blood cortisol and sputum indices in asthmatic subjects treated with or without inhaled corticosteroids may give a clue to this question.

Although differences in severity, level of control of asthma, and inhaled steroid treatment may play a role, other potential technical confounders should also be looked at. We have made our sputum assessments at 8 AM and 8 PM, whereas Panzer et al [5] made them at 8 AM and 4 PM maybe because of the circumstances related to the working hours of laboratory facilities. Further on, we froze our sputum samples at -70°C upon collection both in the morning and in the evening, and worked them out in batches within the next month. Although freezing sputum may bring about minor changes in cell viability, we have no reason to believe that the thawing selectively depletes the samples of certain cell types, which we have studied in the process of developing the protocol. We have already selectively used this approach in a field study [17].

Interestingly, cell viability was higher in the morning samples of all the study subjects. This finding requires special attention, as an association between cellular viability and the reliability of sputum measurements has been demonstrated [18]. A likely explanation may be that airway cells are negatively affected by atmospheric factors (pollutants) through the more vigorous ventilation accompanying daytime activities.

The evening sputum samples of the asthmatic subjects contained relatively more macrophages. As macrophages are the "normal" cell type, which is decreased at the expense of eosinophils and neutrophils in the airways, this may mean that the integrated eosinophil/neutrophil pool is dominating the morning samples and is reduced in the evening ones.

As might be expected, asthmatic subjects had higher ECP values than the normal controls. The healthy subjects had significantly higher ECP levels in the morning than in the evening specimens, whereas the asthmatic subjects had somewhat higher evening values, although not statistically different. This may represent a basic difference in the circadian pattern of the turnover of eosinophils and their products into the airway lumen in health and disease. However, there were no significant correlations between morning/evening differences of eosinophils, ECP, and the other sputum cellular types on one hand, and the serum cortisol levels on the other. Maybe the influence of physiological levels of cortisol on induced sputum cannot be picked up by the design we used in this study.

## Conclusions

The content of sputum exhibits circadian rhythmicity in healthy subjects, which may not be seen in airway inflammatory disease. Identifying the specific features of circadian changes in health and disease will improve our knowledge of the kinetics of the undergoing processes and prevent bias in studies, looking at sputum outcomes around the clock. Freezing of the sputum samples at the time of their collection makes the method of sputum examination much more convenient and applicable.

## End Note

Supported by grant L-622 from the Bulgarian National Research Fund.
